# Assessment of biomechanical behavior of immature non-vital incisors with various treatment modalities by means of three-dimensional quasi–static finite element analysis

**DOI:** 10.1038/s41598-023-44609-2

**Published:** 2023-10-15

**Authors:** Layla Hassouneh, Manal Matoug-Elwerfelli, Taher Al-Omari, Frank C. Setzer, Venkateshbabu Nagendrababu

**Affiliations:** 1 Department of Conservative Dentistry, Faculty of Dentistry, Jordan University of Science and Technology, Irbid, Jordan; 2 College of Dental Medicine, QU Health, Qatar University, Doha, Qatar; 3 Department of Endodontics, School of Dental Medicine, University of Pennsylvania, Philadelphia, PA USA; 4 Department of Preventive and Restorative Dentistry, College of Dental Medicine, University of Sharjah, Sharjah, UAE

**Keywords:** Health care, Medical research, Materials science

## Abstract

The objectives of this study were to evaluate the stress distribution and risk of fracture of a non-vital immature maxillary central incisor subjected to various clinical procedures using finite element analysis (FEA). A three-dimensional model of an immature central incisor was developed, from which six main models were designed: untreated immature tooth (C), standard apical plug (AP), resin composite (RC), glass-fibre post (GFP), regeneration procedure (RET), and regeneration with induced root maturation (RRM). Mineral trioxide aggregate (MTA) or Biodentine^®^ were used as an apical or coronal plug. All models simulated masticatory forces in a quasi–static approach with an oblique force of 240 Newton at a 120° to the longitudinal tooth axis. The maximum principal stress, maximum shear stress, risk of fracture, and the strengthening percentage were evaluated. The mean maximum principal stress values were highest in model C [90.3 MPa (SD = 4.4)] and lowest in the GFP models treated with either MTA and Biodentine^®^; 64.1 (SD = 1.7) and 64.0 (SD = 1.6) MPa, respectively. Regarding the shear stress values, the dentine tooth structure in model C [14.4 MPa (SD = 0.8)] and GFP models [15.4 MPa (SD = 1.1)] reported significantly higher maximum shear stress values compared to other tested models (p < 0.001), while no significant differences were reported between the other models (p > 0.05). No significant differences between MTA and Biodentine^®^ regarding maximum principal stress and maximum shear stress values for each tested model (p > 0.05). A maximum strain value of 4.07E−03 and maximum displacement magnitude of 0.128 mm was recorded in model C. In terms of strengthening percentage, the GFP models were associated with the highest increase (22%). The use of a GFP improved the biomechanical performance and resulted in a lower risk of fracture of a non-vital immature maxillary central incisor in a FEA model.

## Introduction

Endodontic management of immature permanent incisors with non-vital (necrotic) pulps is challenging. These teeth present with an inherent structural weakness due to their thin dentinal walls, a compromised crown-to-root ratio, and a large open apex^1^. Clinically, this makes conventional root canal treatment difficult and leaves the tooth susceptible to fracture, mainly in the cervical area^2,3^. Because of this well-known clinical problem^4^, attention has focused on the reinforcement of these weakened teeth to improve their resilience and, most importantly, increase their long-term survival. Clinically, root fractures can be influenced by several factors such as; the size and shape of the root, the amount and integrity of the remaining tooth structure, crown-to-root ratio, and the occlusion status^3,5^. The mechanical properties of dental materials used for previous restorations or posts, and diseases affecting the structure of dentine, such as osteoporosis could also play a role in tooth/root fracture^5,6^.

The introduction of calcium-silicate materials such as mineral trioxide aggregate (MTA) and Biodentine^®^ as apical plug materials are commonly used with high clinical success rates^7,8^. However, despite the reported short to medium term survival and/or success rates, apical plug techniques do not promote any further quantitative or qualitative increase in root dimensions, resulting in thin, friable roots after the completion of treatment^1^. To overcome the lack of continued root development, regenerative endodontic therapy (RET) has received considerable attention as an alternative biological-based treatment approach^9,10^. For a predictable outcome, the triad of stem cells, a suitable scaffold, and growth factors released from the dentine should be clinically present in an environment free of bacterial contamination^9,11^. Lack of any of the above listed parameters may directly compromise RET outcome^4^. Recently, results of a systematic review and meta-analysis reported, that although previous studies have reported favorable RET survival and success rates, the pooled relative risk revealed no statistical significance between the intervention (RET) and the control (such as apical plug)^12^. They further concluded unreliable outcomes due to high bias and low certainty level of evidence supporting RET in mature and immature permanent teeth with apical periodontitis^12^.

Therefore, from a clinical perspective in cases of unsuccessful RET, alternative treatment options to reinforce a structurally compromised immature tooth, especially in the weak cervical area, should be explored. The use of endodontic posts have been suggested to reinforce these compromised teeth, irrespective of the condition of the clinical crown, including intact or slightly damaged crowns^13^. Clinically, various post types, cements, and coronal restorative materials are available to the treating clinician^14,15^. Glass-fibre posts (GFPs) demonstrate beneficial physical properties, such as a low modulus of elasticity close to that of dentine, resulting in a more favourable stress distribution^16^. This improved stress distribution may lead to a reduced risk of fracture, particularly in the weak cervical area. Clinically, aiding structural reinforcement, reducing polymerisation shrinkage stress thus prevention of/minimising coronal microleakage were regarded as essential parameters for clinical success^14^. Furthermore, as the crown-to-root ratio are compromised in such underdeveloped teeth, which ultimately affect the inserted post length, the mechanical performance of GFPs in these extreme situations is unclear. To date, GFP usage has been mainly reported on mature teeth^17^, bovine immature teeth^16^, with limited data on their application in human immature teeth.

Conducting in-silico studies, mainly finite element analysis (FEA), are an increasingly utilised assessment method and regarded as a useful tool in understanding various biomechanical properties that cannot be examined nor assessed under standard in-vitro experimental models^18^. Within the dental field, in-silico study designs have mainly been performed to gain a deeper understanding of specific biomechanical performance, stress and strain distribution and vulnerable (fracture prone) areas including the tooth and restoration interface^19–21^. Therefore, taking all of the above into consideration and the low certainty of evidence supporting RET^12^ this study was formulated to compare alternative treatment options for immature non-vital teeth. The primary aim of this three-dimensional (3-D) quasi–static linear FEA study, was to assess the biomechanical behavior of an immature permanent maxillary central incisor tooth treated with GFPs compared to an apical plug, resin composite and RET. A secondary aim of this study was to compare the biomechanical behavior of MTA and Biodentine^®^ as an apical or coronal plug material.

## Results

### Maximum principal stress (MPS)

The dentine tooth structure in model C (control model) reported the highest MPS values [90.3 MPa (SD = 4.4)] among all tested models, with a statistically significant difference (p < 0.001) in comparison to the RRM model [MTA: 83 (SD = 4.8) and Biodentine^®^: 82.6 (SD = 4.7)] and GFP model [MTA: 64.1 (SD = 1.7), and Biodentine^®^: 64 (SD = 1.6)]. On the other hand, no significant differences were reported between model C and the AP models [MTA: 88.5 (SD = 4.4; p = 0.24) and Biodentine^®^: 88.2 (SD = 4.4; p = 0.17)]. Overall, statistically significantly lower MPS values were reported in both GFP and RRM models in comparison with AP and RET models (p < 0.05). RC models [MTA: 86.5 (SD = 4.4) and Biodentine^®^: 86.3 (SD = 4.3)] reported significantly higher MPS values compared to GFP models (p < 0.001), but no significant difference was found in comparison to RRM models (p > 0.05). Furthermore, no statistically significant difference in the MPS values between both investigated calcium-silicate materials (MTA and Biodentine^®^) were reported for each of the experimental models (p > 0.05).

**Figure 1:**
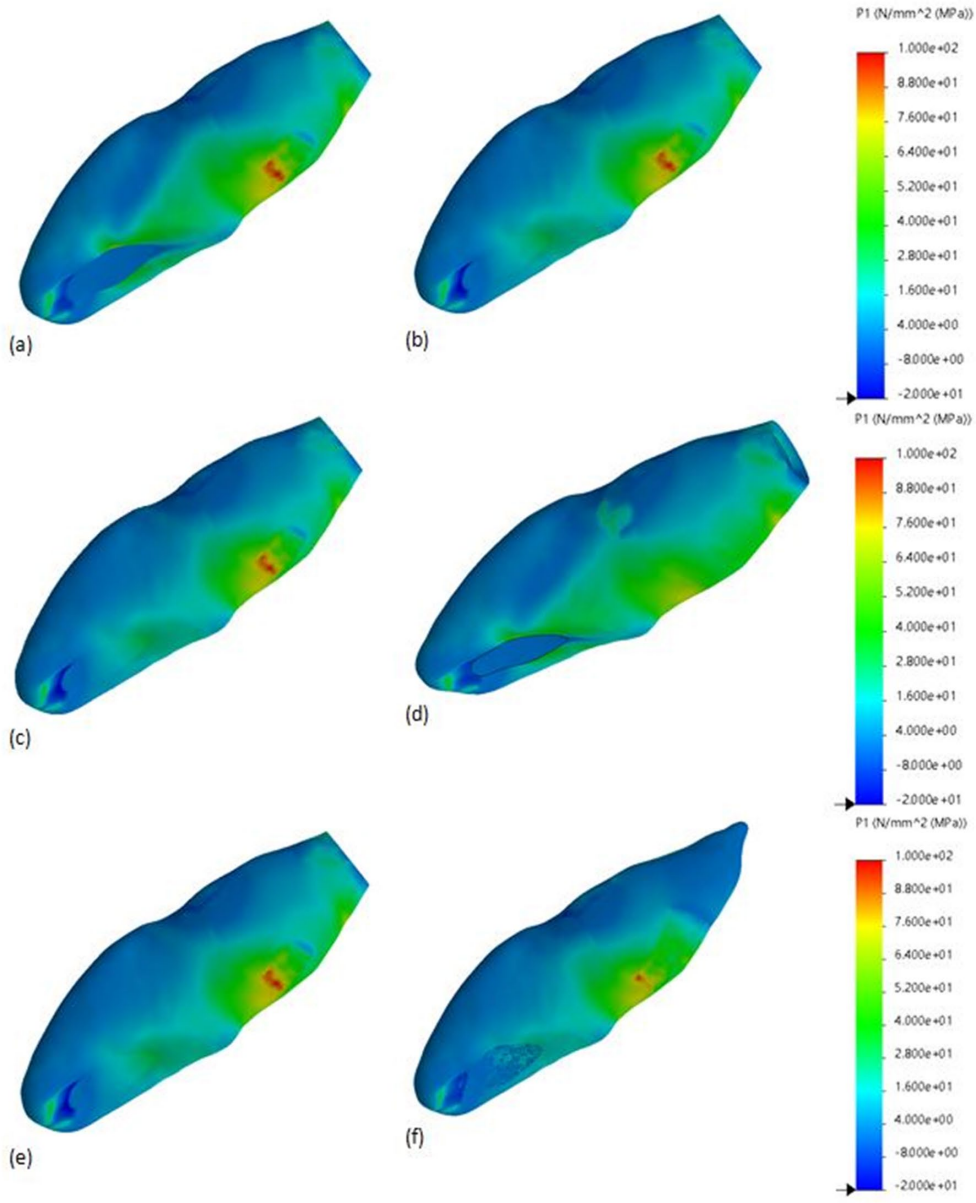
Maximum principal stress distributions (MPa) in control model and experimental models treated using MTA as an apical or coronal plug material. (**a**) Control model, (**b**) apical plug model, (**c**) resin composite model, (**d**) glass-fibre posts model, (**e**) regeneration model, (**f**) regenerative and root maturation model. The blue area corresponds to least stress areas, while red area corresponds to highest stress areas.

**Figure 2:**
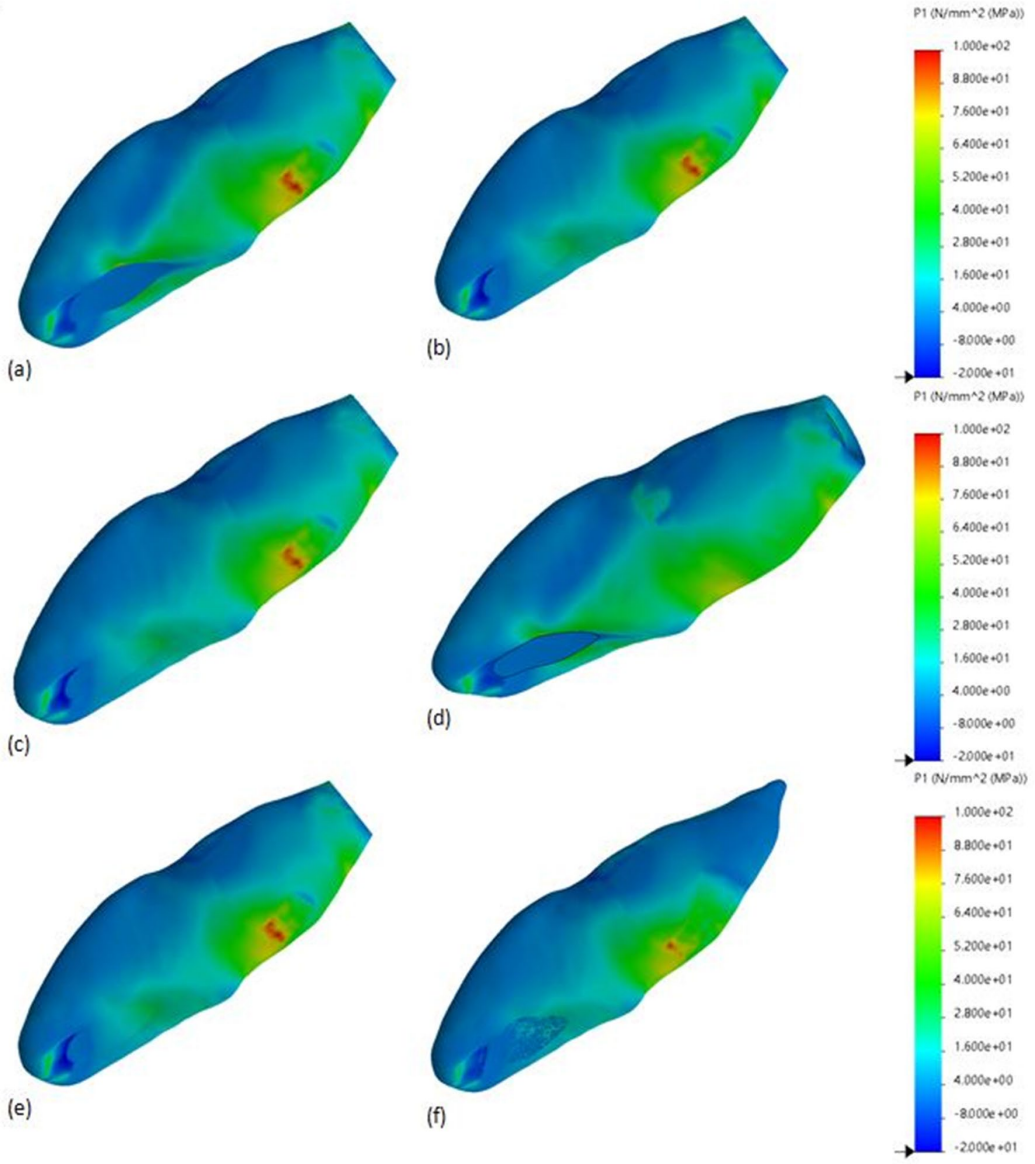
Maximum principal stress distributions (MPa) in control model and experimental models treated using Biodentine^®^ as an apical or coronal plug material. (**a**) Control model, (**b**) apical plug model, (**c**) resin composite model, (**d**) glass-fibre posts model, (**e**) regeneration model, (**f**) regenerative and root maturation model. The blue area corresponds to least stress areas, while red area corresponds to highest stress areas.

Stress patterns in the FEA models revealed that the area of highest stress concentration was located at the cervical root region for all investigated models (Figs. 1, 2). Model GFP showed the most homogenous stress distribution pattern followed by the RRM and RC models, resulting in lower stress concentration at the cervical root region. However, models R and AP had similar stress distribution patterns compared to model C. In terms of material usage, both MTA and Biodentine^®^ resulted in similar stress distribution patterns for each experimented model.

### Shear stress

Regarding the shear stress values, the dentine tooth structure in model C [14.4 MPa (SD = 0.8)] and GFP models [15.4 MPa (SD = 1.1)] reported significantly higher maximum shear stress values compared to other tested models (p < 0.001). Models AP [11.4 MPa (SD = 1.8)], RET [11.5 MPa (SD = 1.7)], RRM [12 MPa (SD = 0.8)], and RC [11.4 MPa (SD = 1.8)], all reported relatively similar maximum shear stress values with no significant difference among them (p > 0.05). No difference in the maximum shear stress values between both investigated calcium-silicate materials (MTA and Biodentine^®^) were reported for each of the experimental models. Shear stress patterns in all FEA models revealed that the areas of highest stress concentration was located coronally at the load application region followed by the mid-root region in the restored models.

### Strain distributions

**Figure 3:**
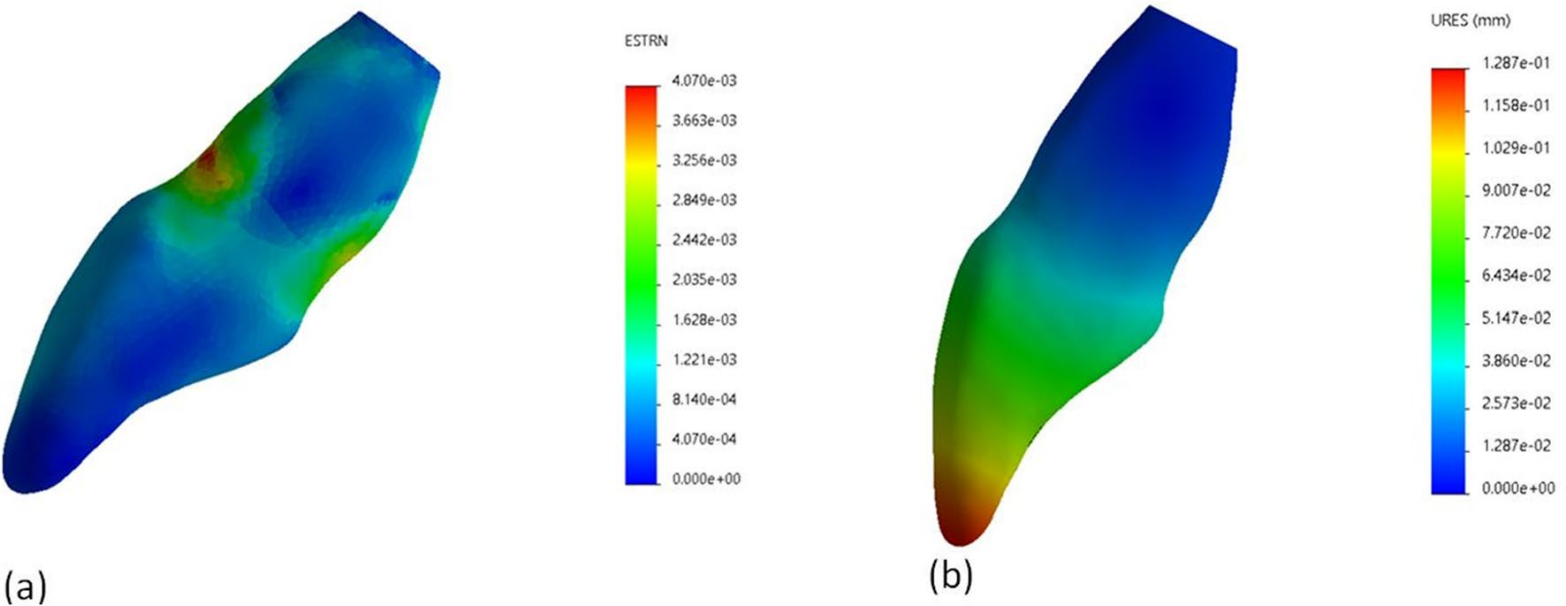
(**a**) An image illustrating strain distribution in control model. (**b**) An image illustrating displacement distribution in control model. The blue area corresponds to least strain/displacement areas, while red area corresponds to highest strain/displacement areas.

Analysis of strain distribution revealed that the tooth structure in all tested models deformed mainly in the cervical and middle regions of the root, while less deformation was recorded in the crown (Fig. 3a). A maximum strain value of 4.07E−03 was recorded in the root dentine below the cemento-enamel junction of model C. The rest of the models recorded a maximum strain value ranging from 2.9E−03 in RRM model to 3.91E−03 in GFP model.

### Displacement

The maximum displacement was reported at the incisal margin for all models. The maximum values of displacement of model C was 0.128 mm (Fig. 3b). The rest of the models recorded a maximum displacement magnitude ranging from 0.121 mm in GFP model to 0.109 mm in RC model.

### Risk of fracture

Results of the risk of fracture were assessed based on the FoS. The FoS values for all tested models were above 1, indicating that all models are safe at the tested load. However, models C (1.063), RET (1.064), and AP (1.084) reported the lowest FoS values, followed by model RC (1.098) and model RRM (1.127), while model GFP (1.267) was associated with the highest FoS values.

### Strengthening percentage

Overall, the results revealed that the highest strengthening percentage was seen in GFP (22%), RRM (5%) and RC (3%) models. The remaining models had a less increase in strength of around 1–2%.

## Discussion

The current study investigated the biomechanical behavior of immature maxillary incisors treated with various treatment modalities and subjected to oblique loading utilising an FEA approach. While regenerative techniques have become commonplace, not every treatment turns out to be successful^12^. In case of an unsuccessful outcome, thin root walls and a wide-open apical foramen may persist, and the tooth remains susceptible to fracture. Our study explored which secondary treatment options would increase fracture resistance, particularly the traditional apical plug procedure reinforcement with a GFP and the traditional apical plug procedure and root filling with gutta-percha.

Posts aid in providing retention for build-ups under definitive restorations, however, do not strengthen a tooth^22^. The length of a post can affect the resistance and stability of a tooth, particularly in cases of root fracture. Proper post placement can help to distribute forces during biting and chewing. An optimal post length is affected by various factors, such as the remaining root structure and morphology, the type and location of an existing fracture, bite forces and occlusion, restorations, and the crown-to-root ratio^5,22^. Post materials can also influence potential reinforcement and fracture prevention. Materials may include metal alloys, fibre-reinforced composites, or zirconia. The elastic modulus of fibre-reinforced composite posts is close to that of dentine, resulting in a lower stress transmission by a fibre post compared to titanium or zirconia^6^. Whether stress transmission and rigidity of a post influence fracture resistance and failure mode of endodontically treated roots with posts has been controversial^23,24^.

In general terms, tooth fractures may occur due to both compressive and tensile forces acting on the tooth structure^25,26^. Compressive forces relate to the application of pressure on a tooth, applied along a tooth's longitudinal axis. Biting or bruxism may exert compressive forces that can lead to fractures^27^. Tensile forces involve the application of tension or stretching forces that pull the tooth structure apart^26^. Tensile stresses may be a result from trauma or direct impact, e.g. from an accident or sports injury. Root fractures from tensile forces may not only be a result of trauma, but also from orthodontic treatment or malocclusion in form of an incorrect bite alignment^28^. Additionally, existing cracks or other defects from existing trauma may provide stress concentration points in the tooth structure. This may cause material fatigue and result in tooth or root fracture with the impact of compressive or tensile forces lower than in an unaffected tooth^26^.

Overall results from this FEA analysis showed that treatment with an apical plug followed by GFP provided the strongest enhancement of the biomechanical behavior. No significant difference between MTA and Biodentine^®^ when used as the material of choice for the apical or coronal plugs (p > 0.05). A maxillary central incisor model for the FEA investigation was selected, as immature maxillary central incisor often loses pulp vitality, e.g., due to trauma, and subsequently require appropriate treatment^9^. The geometrical models used in this study allowed for an accurate clinical simulation and representation of the internal anatomy, as they were based on the scan of a natural maxillary central incisor. However, in deviation from a true clinical scenario, the adhesive layer between tooth structure and a composite restoration (10 µm) was not simulated or meshed in the FEA due to numerical considerations of this ultra-thin adhesive layer. Hence, the tooth structure and composite restoration were considered as one bonded unit, as previously reported^29,30^.

The microstructure and mechanical properties of dentine and bone tissue differ in adults compared to young patients^31–33^. Therefore, dentine and bone tissue parameters for young patients were adopted in this study to achieve a more accurate clinical simulation of immature teeth. In contrast, other FEA studies applied the material properties for dentine and bone structure of adult patients to generate FEA models of immature teeth, which may have impacted the clinical significance for actual immature teeth^19,20,34,35^. Similarly, physical in-vitro studies also used apically modified and instrumented mature teeth to mimic immature teeth during mechanical testing^36–38^. These limitations may have impacted the simulation of immature teeth and provided inaccurate results. However, in regards to FoS calculations, the ultimate tensile and compressive strength values of dentine were adopted from adult patients^39,40^, as there is insufficient data available in the literature for young dentine.

Clinically, during force application, the PDL is thought to exhibits a non-linear elastic behaviour, with considerable deformations, thus violating the basic assumptions of FEA simulations using linear-elastic theory^41^. Although the most accurate PDL model (non-linear, linear-elastic, bilinear, or hyperelastic) is controversial, the linear-elastic theory is well-validated and commonly used in finite element models^42^, including the current study. Despite, previous studies reporting that variable PDL geometry plays in the tooth-PDL-bone complex during mastication in multi-rooted teeth^41^. As the current study involved single-rooted immature tooth under a quasi–static loading and did not involve or attempt to replicate masticatory (dynamic) forces, and due to the comparative nature of this study, such limitations are unlikely to have an effect on the outcome of this study.

Studies have revealed that the clinical fracture of teeth is usually initiated at the dentine especially in the cervical area^43^. Therefore, this study considered dentine as a structure of interest for stress analysis and failure prediction. Due to the relatively low tensile strength of dentine, teeth are more prone to fracture under tensile forces^25^. Thus, this study focused on the analysis of MPS values. The Mohr–Coulomb failure theory was used to analyse the risk of failure in dentine as this theory can be used to predict fracture in brittle materials with a range of compressive and tensile properties^26^. On the other hand, some studies pointed out that visco-elastic and plastic behaviour also exists notably for hydrated dentine or at the dentine/enamel junction^44^. Accordingly, shear stress could also have a meaningful impact and was analysed in the current study.

Due to the inherent structural weakness of immature teeth, the use of endodontic posts has often been suggested to reinforce these compromised teeth, irrespective if a crown was intact or just slightly damaged^13^. However, there is a lack of scientific evidence to support these assumptions. Our study revealed that the use of a GFP with an apical plug resulted in more favourable stress distribution patterns, specifically a lower stress distribution in the cervical region, compared to an untreated immature tooth (model C). These results are in line with previous findings indicating the reinforcing effect of GFPs in immature teeth^37,45^. The fracture resistance of extracted simulated bovine immature mandibular incisors restored with MTA plugs and GFPs were significantly higher than that of teeth restored with MTA only following in-vitro thermocycling for 500 cycles^46^. However, no significant differences in terms of fracture and impact strength were demonstrated between RET, MTA, and fibre posts in an in-vitro study utilising simulated extracted human teeth^47^. The different findings between studies are most likely attributed to multiple reasons, such as experimental design, source of teeth, and the type and size of GFPs. This highlights the need for further research on the clinical usage of GFPs in immature teeth for sound conclusions to be drawn.

The application of a calcium silicate apical plug with gutta-percha in the remainder of the root canal space failed to reveal any noticeable reinforcement of tooth structure compared to the untreated immature tooth (model C). While the use of MTA or Biodentine^®^ as apical and coronal plug material resulted in minimal stress changes, this did not cause any significant changes in the stress distribution patterns for the applied load of 240 N. On the contrary, immature premolar FEA models with MTA plugs and unfilled canals demonstrated increased stress values at the apical and mid-root regions, but reduced stress at the mid-coronal region if the canal was filled with gutta-percha and the access cavities sealed with adhesive composite resin^19^. However, due to the differences in methodology, such as the tooth type and amount of root development, a direct comparison with this study is problematic.

To closely resemble the clinical outcome, model RRM was designed to simulate intra-canal PDL-like tissue, as shown in previous RET histological studies^48^. According to previous FEA studies investigating the biomechanical performance of immature teeth after attempted RET various models were simulated including either the simulation of pulp-like tissue to fill the canal space^19^ or did not specify the type of tissue simulated^20^. Unfortunately, the simulation of PDL-like tissue in relevant mechanical in-vitro studies is challenging and has not been previously tested. Similarly, the simulation of cementum deposition of a 15% increase in the width and 11% increase length of the root canal was based on RET histological studies^49,50^, randomised clinical trials^51–53^, and recommendations from previous FEA relevant studies^20^. As above, the simulation of cementum deposition in mechanical in-vitro studies although challenging, could add significantly to the literature and help support findings from in-silico studies. Therefore, further studies are required in order to investigate the significance of accurate simulation of cementum and PDL tissue in mechanical in-vitro and FEA studies.

Of clinical interest, the results of this study reported that both RRM and GFP models were associated with more favourable stress distribution patterns compared to the clinical standard apical plug treatment (model AP). Therefore, although GFP treatment does not allow for continued root maturation, the ability of the intra-canal post to bind to the surrounding dentinal structure may be beneficial to strengthen these compromised teeth. However, an increase in root length through RET will be clinically beneficial, especially in teeth with minimally developed short roots, in which RET should be considered the first treatment option^9^. The current results also revealed that models representing RET treatment before root maturation (model RET) had similar stress values compared to model C, which indicated that the tooth would remain fragile during the initial period of RET treatment until sufficient root maturation is deposited.

The shear stress analysis in the current study reported smaller values in comparison to tensile stresses, in which the reported maximum shear stress values are all well below the ultimate shear strength of dentine which ranges from 52.7 MPa near the pulp to 76.7 MPa near the dentine-enamel junction^54^. This indicates that the tensile stresses are more likely to influence the risk of tooth fracture. Previous studies reported similar results^25^. However higher shear stresses could be associated with higher risk of loss of retention of the restorative material^43^. In the current study, the GFP model reported higher shear stress at the internal mid-root region compared to other models. This indicates that the risk of loss of retention is higher in the GFP compared to other materials, such as gutta-percha and resin composite. However, it should be noted that dentine-bonding systems could provide bond strengths of up to 30 MPa^55^, which is considerably higher than the maximum shear stress values reported for the GFP model. Further investigations should be conducted to focus on the bonding conditions of GFP’s in immature teeth.

For all investigated groups, the area of highest MPS stress concentration mainly occurred at the cervical root area, which is in accordance with previous studies^20,45^. This indicated that these areas might be more prone to fracture on loading, possibly because the cervical region may act as a fulcrum on masticatory load application. This highlights the importance of preserving a maximum amount of sound tooth structure, especially at the cervical area. Our results also revealed that FoS was above 1 for all investigated models, indicating that all groups were safe under the tested load. However, models C, AP, and RET were associated with the lowest FoS values, indicating weaker roots that are likely to be more prone to fracture with continuous load application. Of clinical interest, models GFP and RRM had highest FoS, indicating safer and less prone to fracture with continuous load application.

Limitations of the current study include the inherent simplifications commonly associated with dental FEA studies such as; the complexity of dental models, load, and boundary conditions. For example, tooth structures such as dentine and enamel are anisotropic, inhomogeneous biomaterials with internal microstructures^56^. However, most FEA studies in dentistry, including the current study, assumed that the above-mentioned structures are isotropic, linearly elastic, and homogeneous^57,58^. Additionally, FEA models assume a well-bonded interface. However, it is known that ideal bonding is difficult to achieve in a clinical scenario due to irregular geometry, any residual material contamination, and shrinkage of commonly used materials^58^. This study assumed isotropic linear elastic properties for the resin cement. However, the complex definition of restoration–adhesive–dentine interfaces would involve the inclusion of cohesive zone models to allow for strength prediction of the adhesive joint^59^. Furthermore, whenever possible, in-vitro experimental validation should be performed to verify the accuracy of FEA results^58^. This was not performed in the current study and is within our future research plans. It should be noted that different material properties of MTA and Biodentine^®^ have been reported in the literature^19,34,45^. This study adopted the mechanical properties of both materials from the best available evidence to date^19,45^, in addition to information provided by the manufacturers (Septodont, Saint-Maur-des-Fossés, France). However, more studies should be conducted in order to reach more decisive information about such materials. Furthermore, due to the difficulty of simulating the complex interfaces between tooth structure and such materials, further in-vitro and clinical studies are required for sound clinical recommendations.

Despite the above-mentioned shortcoming, due to the comparative nature of the study, such limitations are not likely to affect the current results. Strengths of this study include being the first study to conduct a comprehensive comparison of multiple treatment procedures and materials for an immature permanent maxillary central incisor tooth under standardised conditions of an FEA approach. Additionally, to closely replicate the clinical environment, dentine and bone parameters of young patients were used to formulate the current FE analysis. However, for more accurate results reporting further randomised clinical trials are of utmost importance for sound clinical decision making. In conclusion, the application of a GFP in restoring a pulpless immature incisor tooth improved its biomechanical performance in terms of a more homogenous stress distribution pattern and lower risk of fracture. Therefore, clinical usage of GFPs may be considered as an alternative treatment option, especially in unsuccessful RET cases.

## Methods

This study is designed and reported according to the reporting guidelines for in-silico studies using finite element analysis in medicine (RIFEM) version 1.0^18^.

### Development of geometrical models

A precise and anatomically accurate 3-D model of a mature maxillary central incisor was obtained from a Computerised Tomography (CT) scan (I-CATw, Xoran Technologies, Ann Arbor, MI, USA) of a natural tooth obtained from an open online database^60^. The voxel size was 0.25 × 0.25 × 0.25 mm and the planar resolution 640 × 640 voxels per slice. The adopted model geometry was previously verified ^60^. The different tooth structures (enamel, dentine, and pulp) of the adopted model were imported into computer-aided graphic designing software (SolidWorks 2021, Dassault Systems, SolidWorks Corps, Massachusetts, USA) to simulate the experimental groups, as described below. Using previously published data of dentine and bone parameters for young patients, a 1.5 mm thick cortical bone layer and a trabecular bone section were added and connected to the model with soft tissue composed of a 0.2 mm thick periodontal ligament (PDL)^31^. An immature tooth model was developed by modifying the above mature tooth model by reducing the root length and thickness according to a Cvek stage-3 root development (2/3 of root development with an open apex)^61^. The root length of the immature tooth was approximately 3 mm shorter than the original mature tooth, with an apical opening of approximately 1.67 mm^61^. Additionally, the root thickness was reduced by widening the canal space to accommodate for a root-to-canal ratio of 1:1, in a mesio-distal dimension according to Cvek stage-3 root development^61^. The length of the final immature tooth model was 22.2 mm, the crown length was 11 mm, and the root length was 11.2 mm, which indicates, a crown to root ratio of around 1:1. The mesio-distal dimensions of the root at the coronal third, mid-third, and apical third was 5.4 mm, 4.7 mm, and 3.19 mm, respectively. The mesio-distal dimensions of the canal at the coronal third, mid-third, and apical third was 2.7 mm, 2.3 mm, and 1.6 mm, respectively.

### Model designs

**Figure 4:**
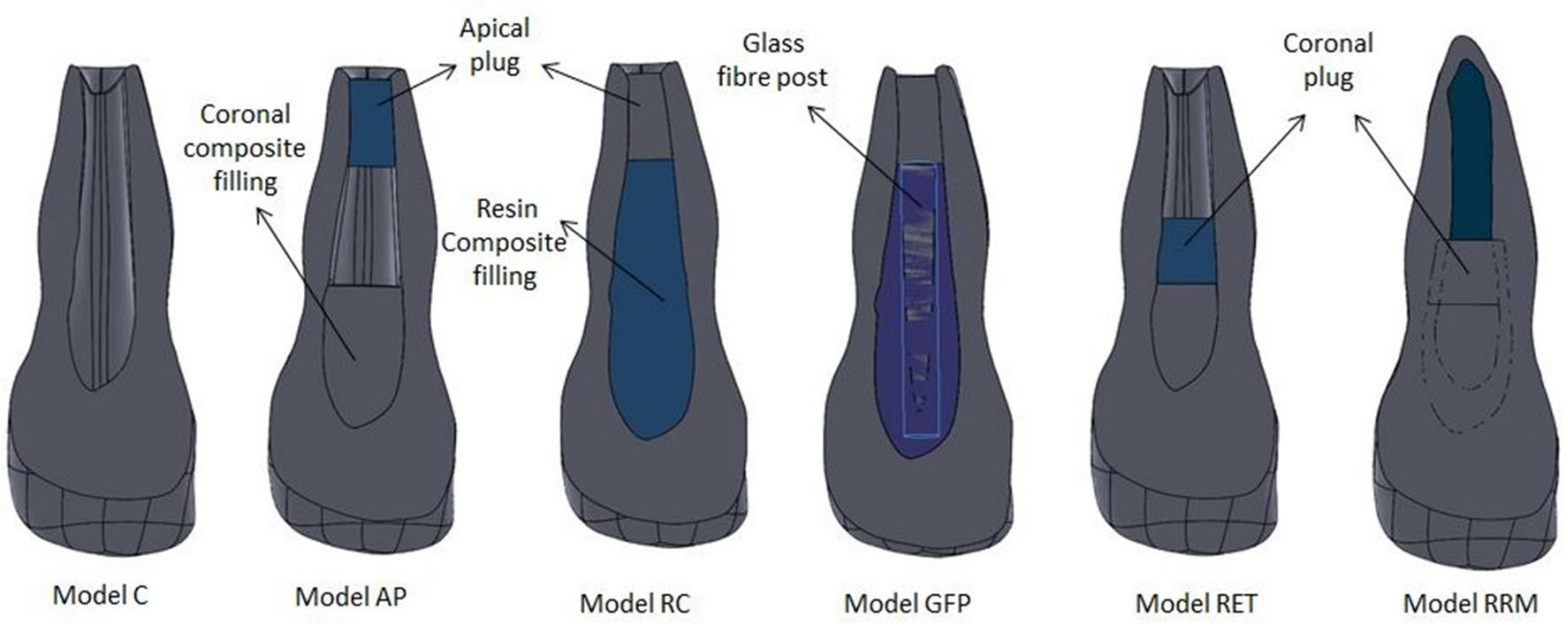
Representative images of the geometry of different experimental models presenting an immature maxillary central incisor (Cvek stage-3 root development [2/3 of root development with an open apex]) with different treatment modalities. *Model C* control model, *Model AP* apical plug model, *Model RC* resin composite model, *Model GFP* glass-fibre posts model, *Model RET* regeneration model, *Model RRM* regenerative and root maturation model.

**Figure 5:**
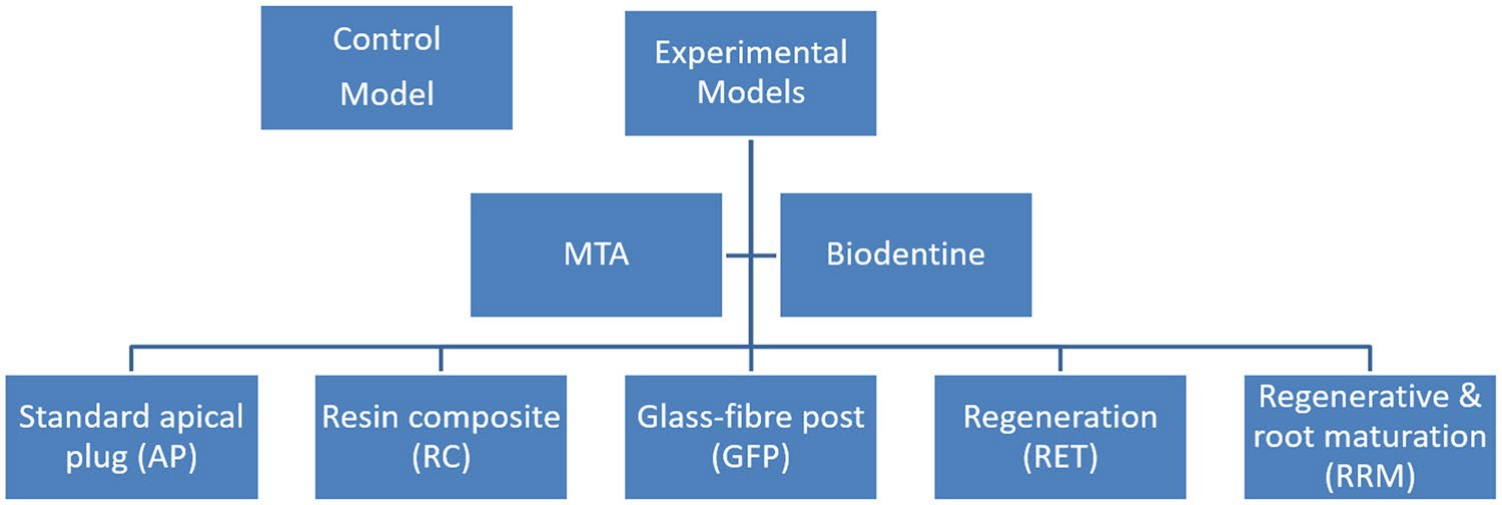
Flowchart representing the different experimental models simulated in the current study.

The model of the immature tooth combined with bone and PDL simulation was considered the control model (model C). Five geometrical models representing an immature maxillary central incisor tooth treated with various clinical modalities were derived from model C (Fig. 4). In total 11 finite element models were analysed as listed below and summarised in (Fig. 5).

- Model I: Control model

Empty root canal space, with no additional treatment.

- Model II and III: Standard apical plug (AP) models

These two models involved standard placement of a 4 mm apical plug material (II: MTA [Pro Root MTA Dentsply, USA], III: Biodentine^®^ [Septodont, Saint-Maur-des-Fossés, France]), the root canal space was filled with gutta-percha (6.2 mm in length) simulating warm vertical obturation followed by a coronal composite restoration (Kuraray America, Tokyo, Japan) 1 mm below cemento-enamel junction.

- Model IV and V: Resin composite (RC) models

These two models involved standard placement of a 4 mm apical plug material (IV: MTA, V: Biodentine^®^), the root canal space was filled with bulk-fill composite (Tetric EvoCeram Bulk Fill, Ivoclar Vivadent, Schaan, Liechtenstein, SonicFill, Kerr Corporation, Orange, CA, USA) followed by a coronal composite restoration 1 mm below cemento-enamel junction.

- Model VI and VII: Glass-fibre post (GFP) models

These two models involved a 4 mm apical plug material (VI: MTA, VII: Biodentine^®^), followed by a 1.2 mm diameter cylinder design to simulate a GFP extending from the apical plug to the coronal composite restoration with a total length of 14.7 mm. Adhesive resin cement (RelyX Unicem, 3 M Espe, Seefeld, Germany) filled the space between the GFP and the root canal, followed by a coronal composite restoration.

- Model VIII and IX: Regeneration (RET) models

These two models stimulated the state of a tooth immediately after RET. No root development or PDL-like tissue was present in the pulpal space. This model only involved a 3 mm coronal plug material (VIII: MTA, IX: Biodentine^®^) placed 1 mm below the cemento-enamel junction, and a coronal composite restoration.

- Model X and XI: Regenerative and root maturation (RRM) models

These two models included simulation of cementum deposition of a 15% increase in the width and 11% increase length of the root canal wall as previously reported^51–53^. The root canal space was simulated to be filled with PDL-like tissue, as reported in the literature^42,62^ (Table 1), a 3 mm root coronal plug material (X: MTA, XI: Biodentine^®^) placed 1 mm below the cemento-enamel junction, and a coronal composite restoration^51^.

**Table 1:** Mechanical properties adopted for simulated tooth tissues and restorative materials.

Tooth structure/material	Young's modulus (*E*, GPa)	Poisson's ratio (*ν)*
Enamel^20,63^	41	0.3
Dentine^32^	15.1	0.3
Cementum^20^	8.2	0.3
Pulp^20,63^	0.003	0.45
PDL^42,62^	0.00427	0.45
Cortical bone^31,33^	10	0.26
Cancellous bone^31,33^	0.5	0.38
MTA^19,45^	11.76	0.314
Biodentine^®^ (Septodont*)^19,45^	22	0.33
Gutta-percha^19^	0.14	0.45
Composite resin^19^	16.4	0.28
Bulk fill resin composite^64^	12	0.25
Resin cement^25^	8.3	0.35
GFP^25^	*E*x = 37	*Vx* = 0.34
	*Ey* = 9.5	*Vy* = 0.27
	*Ez* = 9.5	*Vz* = 0.27

*Gpa* Gigapascal, *PDL* periodontal ligament, *MTA* mineral trioxide aggregate, *GFP* glass-fibre post.

*Septodont, Saint-Maur-des-Fossés, France.

### Finite element analysis

The geometrical models described above were imported into the FEA software for simulation (SolidWorks 2021, Dassault Systems, SolidWorks Corps, Massachusetts, USA). Parabolic tetrahedral solid elements were used in the meshing design of all experimental models. The meshes were generated through a convergence test of 10% strain energy and displacement variation control. The mesh was assessed for element quality and refined in areas of interest. Four FEA test models were used for the convergence test with total nodes and elements in each test model as follows:

Test 1: 36,665, 19,983 nodes and elements, respectively.

Test 2: 75,241, 43,639 nodes and elements, respectively.

Test 3: 160,440, 99,472 nodes and elements, respectively.

Test 4: 293,447, 190,699 nodes and elements, respectively.

**Figure 6:**
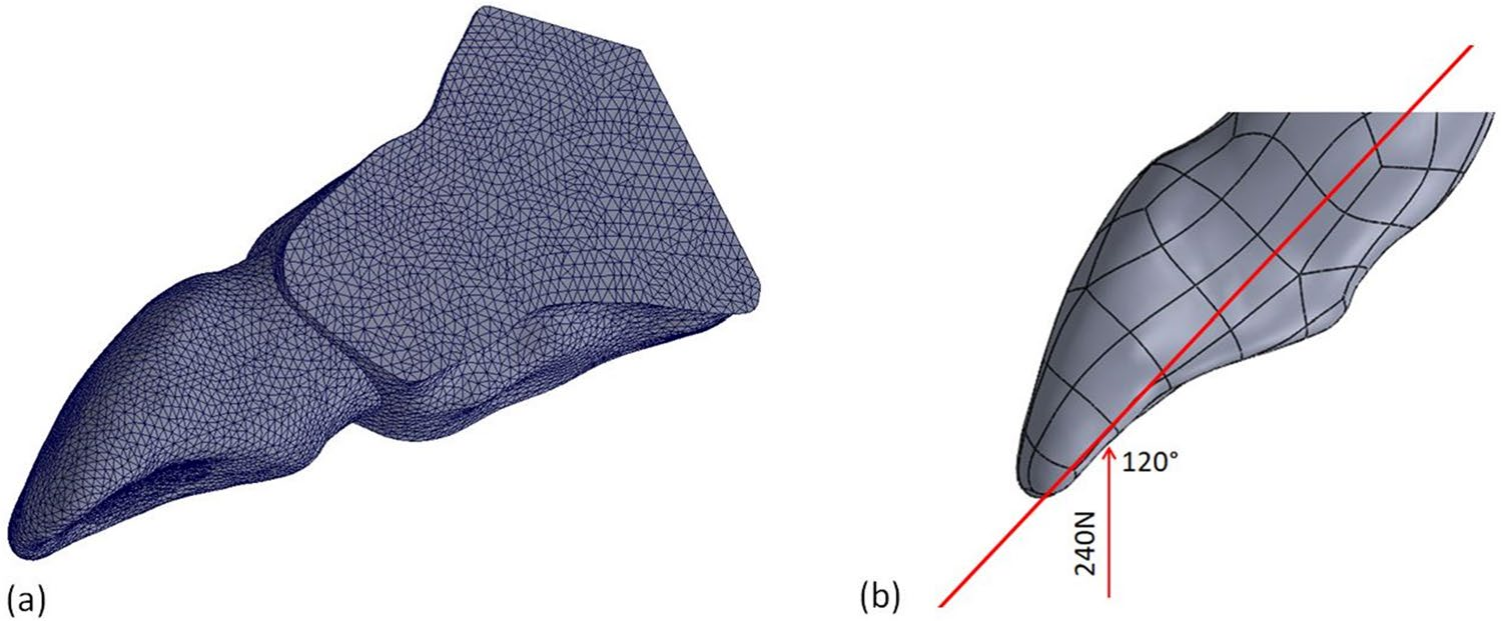
(**a**) An image illustrating the final mesh design of the control finite element model. (**b**) An image illustrating the loading conditions during the FEA simulation. An oblique force of 240 Newton (N) at a 120° angle to the longitudinal tooth axis on the lingual aspect of the incisal edge was applied.

The final number of total elements and nodes in the experimental models varied between 190,699–263,051 elements and 293,447–400,055 nodes. Figure 6a illustrates the mesh design of model C.

The natural tissues (enamel, dentine, cementum, pulp, PDL, bone) and the restorative materials (resin composite, resin cement, gutta-percha, MTA, Biodentine^®^) used in this study were considered linear, elastic, homogenous, and isotropic, with the exception of the GFP, which was considered orthotropic. The material properties were assigned according to the literature as shown in (Table 1). The dentine and bone parameters applied in the current study were adopted from young patients to generate FEA models of close resemblance to immature teeth^31–33^.

Ideal adherence was assumed between all internal materials and tooth structures. The FEA simulated masticatory forces in a quasi–static approach using linear FEA. All models received an oblique force of 240 Newton (N) at a 120° angle to the longitudinal tooth axis on the lingual aspect of the incisal edge (area: 10.78 mm^2^) to simulate masticatory forces (Fig. 6b)^65,66^. In each model, the movement of the mesial, distal and bottom surfaces of the bone were restricted.

### Result analysis

The maximum principal stress (MPS) and the maximum shear stress values on the remaining dentine tooth structure were assessed. To better demonstrate the differences between groups, the 30 highest stress values were selected for quantitative comparison. The mean and standard deviation (SD) of these values were used instead of reporting the highest single peak value, which could be misleading^58^. Data analysis was processed using the Statistical Package for the Social Sciences (SPSS; version 23 IBM Inc., Chicago, USA) with a statistical significance level set at 5% (p < 0.05). The MPS and maximum shear stress results of different models were statistically analysed with Kruskal–Wallis and Dunn post hoc tests. Additionally, the MPS values in the different models were visualised using shade images to demonstrate clinical stress distribution areas. Values of maximum strain and the displacement magnitude in all tested models were also reported.

### Risk of fracture assessment

The Factor of Safety (FoS) theory was used to assess the safety of the models based on the Mohr–Coulomb failure criterion at the remaining dentine tooth structure^67^. The dentine ultimate tensile strength (UTS) and ultimate compressive strength (UCS) were set at 105 Megapascal (MPa) and 298 MPa, respectively^39,40^. The Mohr–Coulomb stress ratio (${\sigma }_{MC}$) was calculated as the following:

$${\sigma }_{MC}=\frac{{\sigma }_{max}}{UTS}+\frac{\left|{\sigma }_{min}\right|}{UCS}$$

Accordingly, the FoS was calculated as the following:

 

$$FoS=\left(\frac{{\sigma }_{max}}{UTS}+\frac{\left|{\sigma }_{min}\right|}{UCS}\right)^{-1}.$$

${\sigma }_{max}$ is the maximum tensile principal stress, and ${\sigma }_{min}$ is the minimum compressive principal stress. A FoS less than 1 indicated that a material at a given location failed locally, while an FoS greater than 1 indicated the safety of the given material at that specific location.

### Strengthening percentage

The strengthening percentage of the tooth structure for each treated model was compared to the untreated immature tooth (control, C). The calculation was based on the below equation, where FoS(i) and FoS(ii) were the FoS values of the model being evaluated and the control model, respectively.

$$Strengthening\, percentage=\left(\frac{FoS\left(i\right)-FoS(ii)}{FoS(ii)}\right)\times 100$$

## Data Availability

All datasets of the current study are available from the corresponding author on reasonable request.
